# The Impact of Heroin Self-Administration and Environmental Enrichment on Ventral Tegmental CRF1 Receptor Expression

**DOI:** 10.1093/ijnp/pyad060

**Published:** 2023-10-21

**Authors:** Ewa Galaj, Eddy D Barrera, Kirk Persaud, Rudolf Nisanov, Apoorva Vashisht, Hindy Goldberg, Nima Patel, Hayley Lenhard, Zhi-Bing You, Eliot L Gardner, Robert Ranaldi

**Affiliations:** Department of Psychological and Brain Sciences, Colgate University, Hamilton, New York, USA; The Graduate Center of the City University of New York, New York, NYUSA; Department of Psychology, Queens College of the City University of New York, Flushing, New York, USA; The Graduate Center of the City University of New York, New York, NYUSA; The Graduate Center of the City University of New York, New York, NYUSA; Department of Psychology, Queens College of the City University of New York, Flushing, New York, USA; Department of Psychology, Queens College of the City University of New York, Flushing, New York, USA; Department of Psychological and Brain Sciences, Colgate University, Hamilton, New York, USA; Neuropsychopharmacology Section, National Institute on Drug Abuse Intramural Research Program, Baltimore, Maryland, USA; Neuropsychopharmacology Section, National Institute on Drug Abuse Intramural Research Program, Baltimore, Maryland, USA; The Graduate Center of the City University of New York, New York, NYUSA; Department of Psychology, Queens College of the City University of New York, Flushing, New York, USA

**Keywords:** CRF, CRF1, heroin, addiction, western blot, RNAscope, self-administration, environmental enrichment, ventral tegmental area, nucleus accumbens

## Abstract

**Background:**

There is a strong link between chronic stress and vulnerability to drug abuse and addiction. Corticotropin releasing factor (CRF) is central to the stress response that contributes to continuation and relapse to heroin abuse. Chronic heroin exposure can exacerbate CRF production, leading to dysregulation of the midbrain CRF-dopamine-glutamate interaction.

**Methods:**

Here we investigated the role of midbrain CRF1 receptors in heroin self-administration and assessed neuroplasticity in CRF1 receptor expression in key opioid addiction brain regions.

**Results:**

Infusions of antalarmin (a CRF1 receptor antagonist) into the ventral tegmental area (VTA) dose dependently reduced heroin self-administration in rats but had no impact on food reinforcement or locomotor activity in rats. Using RNAscope in situ hybridization, we found that heroin, but not saline, self-administration upregulated CRF1 receptor mRNA in the VTA, particularly on dopamine neurons. AMPA GluR1 and dopamine reuptake transporter mRNA in VTA neurons were not affected by heroin. The western-blot assay showed that CRF1 receptors were upregulated in the VTA and nucleus accumbens. No significant changes in CRF1 protein expression were detected in the prefrontal cortex, insula, dorsal hippocampus, and substantia nigra. In addition, we found that 15 days of environmental enrichment implemented after heroin self-administration does not reverse upregulation of VTA CRF1 receptor mRNA but it downregulates dopamine transporter mRNA.

**Conclusions:**

Overall, these data suggest that heroin self-administration requires stimulation of VTA CRF1 receptors and upregulates their expression in brain regions involved in reinforcement. Such long-lasting neuroadaptations may contribute to continuation of drug use and relapse due to stress exposure and are not easily reversed by EE exposure.

**Significance Statement**

Chronic heroin use affects stress-related brain systems contributing to the development of heroin use disorder, a chronic and relapsing medical condition. To better treat heroin use disorder, knowledge on these neuroadaptations would be beneficial. In the current study we used an animal model of heroin use disorder to understand how chronic heroin intake affects the expression of corticotropin-releasing factor sub-type 1 (CRF1) receptors, part of the brain’s stress system. We found upregulation of these receptors in the brain’s key addiction-associated regions of heroin self-administering rats. These adaptations appear to be robust and long-lasting as environmental enrichment, as a behavioral intervention, failed to reverse them.

## INTRODUCTION

The role of corticotrophin releasing factor (CRF) in substance use disorder has received significant attention (Koob, 2010; Roberto et al., 2017; Simpson et al., 2020; Baidoo and Leri, 2022). Much of this is in relation to the stress-related factors of substance use disorder, including lapses and relapses to drug use (Contarino and Papaleo, 2005; Haass-Koffler and Bartlett, 2012; Tunstall and Carmack, 2016; Pleil and Skelly, 2018; Baidoo and Leri, 2022). Systemic administration of CRF1 receptor antagonists decreases heroin self-administration under long- but not short-access conditions in rats (Greenwell et al., 2009; Park et al., 2015); it blocks withdrawal symptoms (Baidoo and Leri, 2022) and attenuates stress-induced reinstatement of heroin seeking (Shaham et al., 1998). Chronic drug intake produces neural adaptations in brain regions associated with drug reward, some of which also house drug abuse–related stress mechanisms. It is established that CRF receptor stimulation plays a strong role in stress-induced reinstatement of cocaine seeking and CRF receptor expression undergoes changes as a function of chronic cocaine intake (Wise and Morales, 2010). However, the role of CRF receptors, which are expressed in several key brain areas involved in reward, reinforcement, and drug seeking (Korosi et al., 2006), has not been fully studied.

To date, it is not known whether chronic heroin self-administration (IVSA) leads to upregulation of CRF1 receptors in the ventral tegmental area (VTA), nucleus accumbens (NAc), substantia nigra (SN), prefrontal cortex (PFC), insula, and dorsal hippocampus (dHippo)—the brain regions implicated in opioid self-administration and relapse (Vaccarino et al., 1985; Chang et al., 1997; Chen et al., 2017; Wang et al., 2018; Galaj et al., 2020b; Joshi et al., 2020; Bossert et al., 2023). We were particularly interested in identifying cell type–specific expression of CRF1 receptors in the VTA and assessing their potential upregulation after chronic heroin exposure. Previous studies showed that there is a unique VTA CRF-glutamate-dopamine interaction involved in addiction (Wang et al., 2005; Wise and Morales, 2010). VTA CRF1 receptors are known for their involvement in cocaine seeking (Blacktop et al., 2011; Vranjkovic et al., 2018), and prior exposure to opioids can cause long-lasting potentiation of glutamatergic-mediated activation of VTA dopamine neurons (Saal et al., 2003). Optogenetic stimulation of CRF-containing neurons in the central amygdala leads to greater break points for cocaine and a significant increase in cFos expression in the VTA (>200%), and stimulation of NAc CRF neurons leads to 175% increases in VTA cFos expression (Baumgartner et al., 2022), suggesting a unique CRF-VTA interaction plays a critical role in drug IVSA.

Acute and chronic experimenter-administered morphine increases AMPA receptor expression in the VTA (Lane et al., 2008), whereas prenatal morphine exposure leads to persistent downregulation of VTA AMPA and NMDA receptors (Wu et al., 2018). Others have shown that morphine delivered by subcutaneous pellet implantation has no effect on VTA AMPA receptor expression, but intermittent subcutaneous administration of escalating doses elevates AMPA subunit, GluR1 levels in the VTA (Fitzgerald et al., 1996). Thus, the precise impact of heroin IVSA on the complexity of CRF-dopamine-glutamatergic interaction in the VTA has not been established.

Growing evidence supports that certain drug- or alcohol-induced neuroadaptations within the mesocorticolimbic system can be prevented or reversed by environmental enrichment (EE), a behavioral intervention that provides physical, social, and/or sensory stimulation (Solinas et al., 2010; Galaj et al., 2020a; Marrero-Cristobal et al., 2022). Our group and others have shown that EE, when administered after cocaine or heroin IVSA, can significantly facilitate abstinence (Peck et al., 2015; Ewing and Ranaldi, 2018; Barrera et al., 2023) and reduce reinstatement of drug seeking (Thiel et al., 2010; Ranaldi et al., 2011; Galaj et al., 2016; Barrera et al., 2021; He et al., 2022). Similarly, EE introduced after conditioning can reduce the expression or reinstatement of ethanol- (Li et al., 2015), heroin- (Galaj et al., 2016), and cocaine- (Solinas et al., 2008; Chauvet et al., 2009) conditioned place preference. Thus, EE introduced during forced or voluntary abstinence in animals can reduce incentive motivation to seek and take drugs and reverse drug-induced neuroadaptations (Solinas et al., 2010; Galaj et al., 2020a; Marrero-Cristobal et al., 2022). Here we investigated whether EE can reverse upregulation of CRF1 receptors in the VTA and alter AMPA GluR1 subunit and dopamine reuptake transporters (DAT) in rats self-administering heroin.

Using a multifaceted approach, we tested the hypotheses that VTA CRF1 receptors play a critical role in heroin IVSA and as a result of chronic heroin intake, these receptors, particularly on dopamine neurons, undergo neuroadaptations along with AMPA GluR1 receptors. We also investigated whether dysregulation of CRF1 receptors would be evident in other regions (insula, dHippo, NAc, PFC, SN) involved in heroin IVSA. Finally, we investigated whether EE, which we have repeatedly shown can significantly attenuate various kinds of drug-seeking or related behaviors, can also reverse any drug-induced neuroadaptations that we might observe here. Thus, in Exp. 1, we locally blocked CRF1 receptors in the VTA to determine their causal role in heroin IVSA. In Exp. 2 and 3, we assessed whether intra-VTA antalarmin affect food self-administration and locomotor activity. In Exp. 4, we used RNAscope in situ hybridization (ISH) to identify cell type–specific expression of CRF1 receptor mRNA in the VTA and assessed their expression after chronic heroin IVSA. We also investigated the impact of heroin IVSA on the interaction between VTA CRF1, AMPA GluR1, and the dopamine system. In Exp. 5, using a western-blot assay, we confirmed our findings and explored other brain regions in which CRF1 receptor protein expression might be affected by heroin IVSA. Our regions of interest included the VTA, NAc, PFC, insula, SN, and dHippo. Lastly, we tested if EE could significantly reverse some of the neuroadaptations observed in the VTA using the RNAscope assay.

## METHODS

This study was carried out in accordance with the guidelines established by the Guide for the Care and Use of Laboratory Animals (Institute of Laboratory Animal Resources on Life Sciences, National Research Council, 2011) and was approved by the Queens College Institutional Animal Care and Use Committee.

### Animals

The subjects in these experiments were male Long Evans rats (n = 43) taken from our in-house colony from breeders purchased from Charles River (Kingston, NY, USA). The rats were 3 months old and weighed between 350 and 450 g at the time of surgery. Postsurgery rats were individually housed on a reversed 12-hour-light/-dark cycle (lights off at 7 am) in temperature- and humidity-controlled rooms. All rats had access to food (LabDiet chow) and water at all times, except during daily 3-hour experimentation sessions. All experimental procedures were conducted during the rats’ active period (dark cycle).

### Drugs

Heroin was a gift from the NIDA Drug Supply Program and was dissolved in saline. Antalarmin (a CRF1 receptor antagonist) was purchased from Tocris Bio-Techne (Minneapolis, MN, USA) and dissolved in a saline solution consisting of 10% dimethyl sulfoxide (DMSO) and 15% Tween 80. RNAscope probes and fluorescent multiplex kits were purchased from Advanced Cell Diagnostics (Newark, CA, USA).

### Surgery

Before surgery, each rat was injected with atropine (0.05 mL of 0.4 mg/mL, i.p.) to prevent mucus build-up and anesthetized with sodium pentobarbital (65 mg/kg, i.p.). The scalp of the rat was shaved, cleaned with Betadine, and an ophthalmic ointment (Paralube Vet ointment) was applied to the eyes to prevent corneal drying. During and after surgery the rats were placed on heating pads to prevent hypothermia.

For catheterization, a small incision was made to the right of the midline of the neck. The jugular vein was exposed and a silastic catheter (Dow Corning, Midland, MI, USA) was inserted so that its tip reached the entrance of the right atrium. The catheter was secured to the vein with silk sutures, and its free end was fed subcutaneously around the back of the neck to exit through the scalp incision. Next, the catheter was connected to a bent 22-gauge stainless-steel tube that served as a connector between it and the drug line.

For intracranial cannula placement, the rat’s head was fixed in the stereotaxic apparatus. Two holes were drilled through the skull above the VTA, and 2 stainless-steel guide cannulae (23 gauge/16 mm long) were implanted bilaterally into the VTA using the following coordinates: AP −5.6, ML ±2.2, and DV −8.15 at 10° angle away from the midline (Paxison and Watson, 2013). The cannulae and catheter connector were permanently fixed to the skull using Gorilla superglue and dental acrylic anchored to 4 stainless-steel screws screwed into the skull while the rat was still in the stereotaxic apparatus. Obturators, extending 1 mm beyond the cannulae, were inserted into the cannulae to prevent blockage and remained there at all times except during microinjections. After surgery, the rats were administered carprofen (5 mg/kg, s.c.) and were placed on a heating pad. They were closely monitored during postsurgery recovery and experimentation periods. To maintain its patency, the catheter was filled with 0.05 mL of heparin (200 U/mL of saline) immediately after surgery and daily thereafter. The rats were given 2 or 3 days of recovery before i.v. drug self-administration began.

### Operant Conditioning Chambers

Heroin and food self-administration took place in 8 Med-Associates operant conditioning chambers, each measuring 28 cm × 23 cm ×  30.5 cm (H ×  W ×  L) and placed in a ventilated, sound-attenuating cubicle with an operating fan to mask outside noise. Each chamber was equipped with 2 retractable levers, a white light above each lever, and a drug line consisting of a metal tether covering a polyethylene tubing, which, through a fluid swivel, was connected to a syringe pump (Razel, 3.33 rpm) loaded with a 20-mL syringe.

### Environmental Enrichment

In Exp. 4, rats were housed in enriched or non-enriched environments after heroin self-administration. Each enrichment cage measured 36 cm ×  66 cm ×  41 cm and was equipped with beta chip bedding, a running wheel, a 10-cm diameter tunnel, wooden blocks, and paper towel rolls. Three additional objects (e.g., jingly ball, mirrored bowl, glass mug, paper ball, plastic blocks, sock, rock, leaf, dog chew, and stuffed animals) were replaced daily with different objects.

### Procedure

#### Exp. 1. Assessing the Impact of Intra-VTA Antalarmin on Heroin Self-Administration

Seven rats were trained to self-administer heroin under a fixed ratio 1 (FR1) schedule of reinforcement during daily 3-hour sessions. A press on the active lever activated the white light cue above the active lever and the pump delivering 0.125 mL of heroin solution (0.05 mg/kg/injection) over 4.5 seconds. Each injection was followed by a 20-second time-out during which the light cue remained on and additional lever presses were counted but were not reinforced. Pressing on the inactive lever was counted but had no consequences. Rats continued IVSA training for 7–10 days until reaching the criteria of stable responding (defined as 3 consecutive sessions where the total number of infusions taken per session did not vary by more than ±10% of the mean of the 3 sessions and with no ascending or descending trends). Next, rats were tested with antalarmin. Before the test, obturators were removed and microinjectors, extending 1 mm beyond the guide cannulas, were inserted into the cannulas. Bilateral microinjections of antalarmin, a CRF1 receptor antagonist [0 (n = 7), 1 (n = 8), 2 (n = 6), and 4 (n = 6) µg/0.5 µL/side] were delivered over 60 seconds using a 10-µL Hamilton syringe and the pump. Microinjectors were kept in place for an additional 2 minutes to allow the drug to diffuse. Next, the obturators were inserted back into the guide cannulae, and the rats were placed into the operant conditioning chambers where they self-administered heroin, as previously. Each rat was tested with 4 doses in a random order and with 4–5 days of self-administration sessions between tests. For each test, a new baseline with stable responding was established.

#### Exp. 2. Assessing the Effect of Intra-VTA Antalarmin on Food Self-Administration

Given our findings that VTA CRF1 receptors play a critical role in heroin IVSA, we then assessed the involvement of these receptors in nondrug rewards such as food. A separate group of rats (n = 7) underwent a surgical procedure involving implantation of bilateral cannula into the VTA. After recovery, rats were food restricted to reduce their body weight to 85% of their free-feeding weight and introduced to sugar food pellets in their home cages. Next, rats were trained to press the active lever for food during daily 1-hour sessions. The inactive lever produced no consequences. Once rats achieved a stable baseline (with 3 days of similar responding within 10% variation), they were repeatedly tested under the intra-VTA antalarmin treatment. Before testing sessions, antalarmin was injected directly into the VTA at doses: 0, 1, 2, or 4 µg/0.5 µL/side, and rats were allowed to press the lever for food. All rats were tested with 4 doses, chosen in a random order and with newly established baseline between the test sessions.

#### Exp. 3. Assessing the Impact of Intra-VTA Antalarmin on Locomotor Activity

To rule out the possibility of potential nonspecific effects of intra-VTA antalarmin on behavior, we conducted an additional experiment involving locomotor activity. Briefly, separate group of rats (n = 8) had cannula implanted into the VTA. After recovery, basal locomotor activity was measured for 2 hours for 4 days. Rats then were microinjected with antalarmin (0, 1, 2, or 4 µg/0.5 µL/side) directly into the VTA before testing sessions. For each rat, the order of tested doses was randomly chosen.

### Histology

After completion of behavioral testing, all rats were deeply anesthetized with sodium pentobarbital and perfused with 200 mL of saline followed by 100 mL of 4% paraformaldehyde and decapitated. The brains were removed and stored in 4% paraformaldehyde overnight, followed by transfer to 30% sucrose solution before being sliced in coronal sections and inspected for cannulae implantation and injection sites. Only rats with bilateral cannula placements in the VTA were included in data analyses.

#### Exp. 4. Assessing the Impact of Heroin Self-Administration on CRF1 and AMPA Receptor Expression in the VTA

To assess whether heroin self-administration upregulates CRF1 and AMPA receptors in the VTA, we used RNAscope ISH to target *CRF1*, *AMPA*, and *DAT* mRNA in the VTA of saline– and heroin–self-administering rats (n = 4 per group). Following 15 days of saline or heroin self-administration (as described in Exp. 1), brains were extracted and immediately submerged in 2-methylbutane to be stored in −80°C until ready for use. Coronal sections of rat midbrain were collected at 16 μm thicknesses and mounted onto Superfrost Plus slides. Sections were dehydrated in graduated ethanol phosphate buffered saline (PBS), 50%, 70%, and 100% ethanol) and then processed strictly according to the RNAscope ISH multiplex fluorescent protocol, provided by Advanced Cell Diagnostics. Briefly, using RNAscope Reagent Kit 1 (ACD-Biotechne, Newark, CA, USA), the midbrain sections were incubated first in Protease-Pretreat 4 solution (RT, 20 minutes), rinsed twice in dH_2_0, and then treated with *Rn-Crhr1* (Cat # 318 911; encoding CRF1 mRNA), *Rn-Gria1* (Cat # 540 891-C2; encoding AMPA GluR1 mRNA), and *Rn-Slc6a3* (Cat # 319 621-C3, encoding DAT mRNA) (ACD-Biotechne) for 2 hours at 40°C. After double-rinsing in wash buffer, the sections were treated with AMP 1 (30 minutes, 40°C), AMP 2 (15 minutes, 40°C), AMP 3 (30 minutes, 40°C), and AMP 4 Alt A (15 minutes, 40°C) and double-rinsed in PB between each amplification step. Next, a drop of 4’,6-diamidino-2-phenylindole( DAPI) was added to each slide, followed by fluorescent mounting medium (Fluoro-Gel, Electron Microscopy Science) and coverslip.

Images were taken on a Leica SP-5 confocal microscope using identical settings from 3 sections per rat at 40× magnification from the left and right VTA. Z-stacks (approximately 12 microns) were taken at a resolution of 512 × 512 pixels. DAPI-positive cells expressing CRF1, AMPA, and DAT mRNA, and the number of CRF1, AMPA, and DAT puncta per cell were identified and counted using the open source quantification software Cell Profiler (Carpenter et al., 2006; Erben and Buonanno, 2019) and analyzed with a custom-made R script. We also quantified the density (puncta/area [µm^2^]) for each of our targets.

#### Exp. 5. Assessing the Impact of Heroin Self-Administration on CRF1 Expression in Different Limbic Regions

To assess the impact of heroin self-administration on the expression of CRF1 receptors in key addiction-related brain regions (PFC, insula, NAc, VTA, dHippo, SN, amygdala), we trained different groups of rats (n = 4 per group) to self-administer saline or heroin for 15 days and processed their brains using western-blot assay. After completion of IVSA training, rats were decapitated, and brains were rapidly frozen by brief submersion in cold 2-methylbutane and then stored in −80°C until further use. Brain regions of interest, the PFC, insula, NAc, VTA, dHippo, SN, were carefully cut out from 100-μm coronal sections collected with a cryostat at −20° C. Each region was verified using the Paxinos and Watson brain atlas (Paxison and Watson, 2013). Tissues were homogenized in 150 μL of lysis RIPA buffer, containing protease inhibitor (Thermo Scientific, Waltham, MA, USA) and centrifuged at 14 000 rpm at 4°C for 10 minutes. Protein concentration was then determined using the Biorad microplate reader and compared with bovine serum albumin standards, using the Pierce kit (Thermofisher). Individual samples were diluted in dithiothreitol (5X DTT; Thermofisher) and LDS-sample buffer (4× LDS) to achieve a final concentration of 2 μg/μL. Samples then were heated at 95°C for 5 minutes, and 15 μL of each sample was loaded onto Novex SDS-PAGE (8%–16% Tris-glycine sodium dodecyl sulfate polyacrylamide) gels (Invitrogen, Carlsbad, CA). Proteins were separated based on their molecular weight using electrophoresis at 150 V constant (90 minutes) and transferred to polyvinylidene difluoride membrane (60 minutes) (Invitrogen, Carlsbad, CA). After 1 hour, the membranes were rehydrated in methanol, rinsed in water and 3 times in PBS, and incubated in 5% nonfat/PBS 1X blocking. Next, membranes were incubated overnight in goat anti-CRF1 (1: 1, 000; Abcam; cat # AB77686) and mouse anti-Beta-actin (1: 1, 000; Abcam; cat # AB8227) primary polyclonal antibodies. Membranes were then rinsed 3 times in PBST and once in PBS for 5 minutes and incubated in donkey anti-mouse lgG H&L (HRP) (1: 20 000; Abcam; cat # AB205724) and donkey anti-goat lgG H&L (HRP) secondary antibodies for 2 hours. Following this, membranes were rinsed 3 times in PBST and once in PBS and treated with enhanced chemiluminescence (SuperSignal West Pico Plus Substrate, Thermo Fisher, MA; cat #34 580) for 5 minutes. Images were taken using a Biorad ChemiDoc XRS+ Imager.

#### Exp. 6. Assessing the Impact of EE on Heroin-Induced CRF1 Neuroadaptations in the VTA

In this experiment, we assessed whether 15 days of EE can reverse the upregulation of CRF1 receptors caused by chronic heroin self-administration. Eight rats self-administered heroin under the same conditions as described in experiment 1. After 15 days of heroin self-administration, rats were randomly distributed into EE or non-EE groups (n = 4 per group) and transferred to the corresponding housing where they remained for the remainder of the experiment (15 days). At the end of the experiment, rats were decapitated and brains were extracted and frozen by brief submersion in cold 2-methylbutane and then stored in −80°C until further use. Midbrain sections were processed for *CRF1*, *AMPA GluR1*, and *DAT* mRNA in the VTA, using the RNAscope ISH assay, as described in Exp. 2.

### Data Analysis

For all experiments, total infusion data within daily 3-hour sessions were collected. In Exp. 1, individual baseline infusions were defined as the average across 3 consecutive stable sessions before each test. In Exp. 4–6, total infusions from 15 sessions of drug self-administration were analyzed. Separate 2-way mixed factorial ANOVAs (Exp. 1: dose × phase, Exp. 2–4: session × group) were used. Significant interactions were followed by tests of simple effects (of dose at each phase level, in Exp.1 or of groups at each session level, in Exp. 2–4), and main effects were followed up by Bonferroni post hoc tests (*P* < .05). For Exp. 2 involving food self-administration, active and inactive lever presses was analyzed with repeated-measures 1-way ANOVAs. For Exp. 3. involving locomotor activity, a 2-way repeated measures ANOVA (time × dose) was used to analyzed distance travelled (cm). For RNAscope data, for each rat the average number of CRF1, AMPA, and DAT puncta per cell was calculated with a custom-made R script. We also analyzed the average number of CRF1 or AMPA puncta per DAT cell and their densities. These data were analyzed by separate independent *t* tests. Western-blot data was analyzed using Bio-Rad Imager software and for each brain region, groups (saline vs heroin) were compared using separate independent *t* tests. All data are presented as means and SEM.

## RESULTS

### Exp. 1. Intra-VTA Antalarmin Reduces Heroin Self-Administration

**Figure 1.**
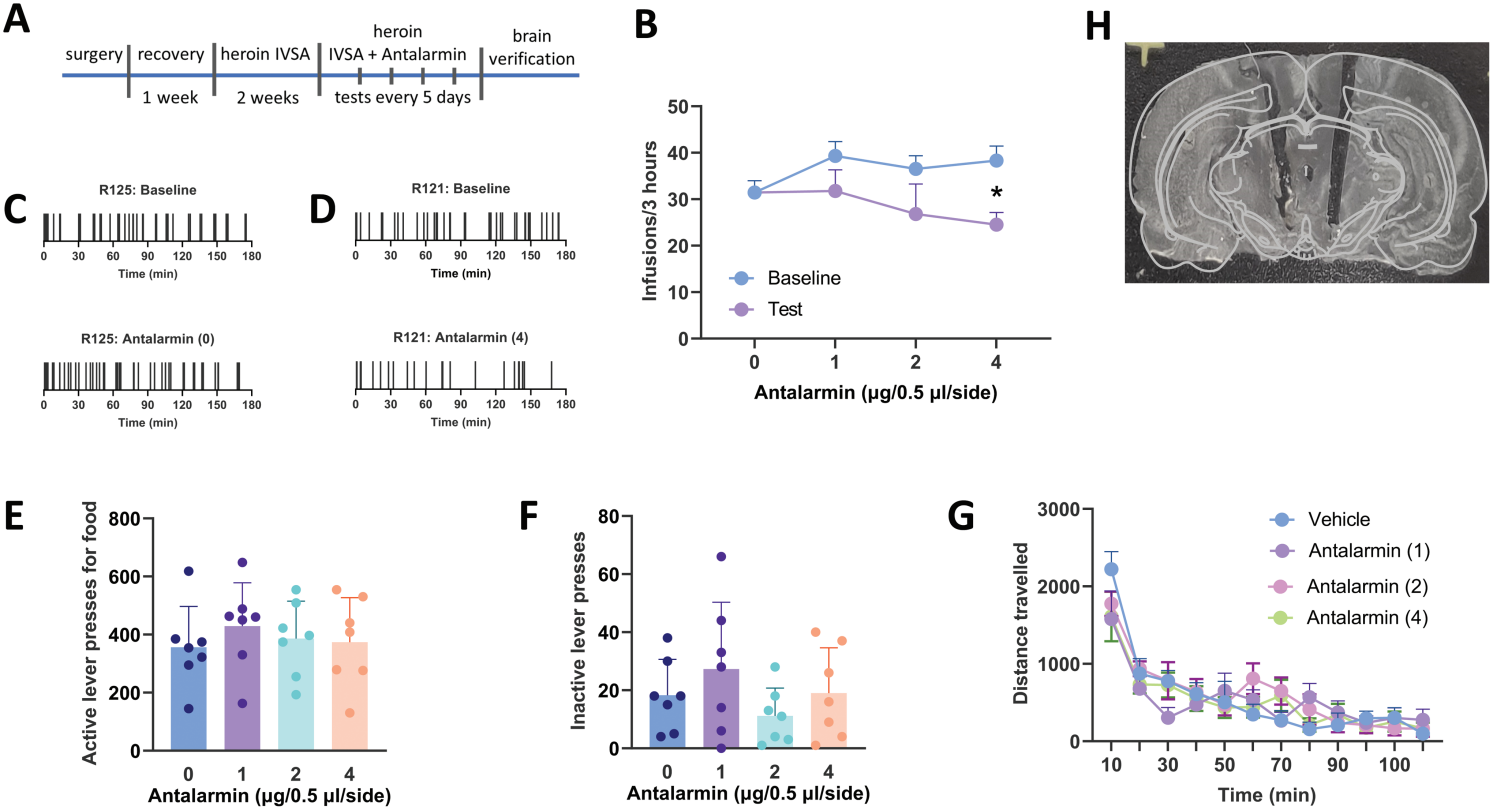
A–H show the data from Exp. 1 investigating the role of ventral tegmnetal area (VTA) corticotropin-releasing factor 1 (CRF1) receptors in heroin self-administration (IVSA). (A) the timeline for behavioral experimentation involving intra-VTA infusions of antalarmin (a CRF1 receptor antagonist) during heroin IVSA. (B) Figure with average means of heroin infusions earned during 3 hour- IVSA sessions in the absence or presence of intra-VTA antalarmin. Pharmacological blockade of VTA CRF1 receptors resulted in a significant reduction in heroin intake; *P < .05. (C) A representative event record of a rat self-administering heroin during a baseline (top panel) and test session with antalarmin vehicle. The pattern of heroin IVSA remained similar across the sessions. (D) A representative event record of a rat self-administering heroin during a baseline and test session with 4 µg/0.5 µL/side of antalarmin, showing heroin intake was reduced during the test with antalarmin. (E) Figure with average numbers of active lever presses for food during 1-hour food self-administration sessions in the presence of intra-VTA antalarmin, indicating no significant changes in food-reinforced lever pressing. (F) The average numbers of inactive lever presses during food self-administration, indicating no effect of antalarmin on nonreward-related behavior. (G) Figure with the average total distance travelled during 2 hours in the presence of intra-VTA antalarmin, indicating that antalarmin had no impact on nonspecific behavior. (H) representative image from cannula placement verification conducted after behavioral assessment.

Although CRF1 receptors are known to play a role in opioid addiction, direct evidence indicating VTA CRF1 receptors as key players in heroin self-administration is still lacking. To address this gap in knowledge, we injected different doses of antalarmin into the rat VTA to block CRF1 receptors before heroin IVSA test sessions. Fig. 1A shows a timeline of behavioral experimentation. Rats that showed a stable baseline of responding were tested with different intra-VTA doses of antalarmin (0, 1, 2, and 4 µg/0.5 µL/side). The 4-µg dose caused a significant reduction in heroin IVSA (Fig. 1B). This observation was confirmed by a 2-way ANOVA that revealed a dose × phase interaction (F_3,24_ = 3.09; *P* = .046). Tests of simple effect of phase at each dose revealed significant differences for the 4 µg (F_1,5_ = 8.50; *P *=* *.033) but not the 0, 1-, or 2-µg doses (*P *> .05). Individual event records indicated that rats maintained stable responding during the baseline (Fig. 1C-D) and when tested with vehicle (Fig. 1C bottom) but not after treatment with the 4-µg dose (Fig. 1D, bottom). Antalarmin reduced heroin IVSA, resulting in irregular patterns of responding. After completion of behavioral testing, we confirmed cannula placement and included into our analysis only data from rats with accurate bilateral placements. Fig. 1H shows a representative brain image with bilateral VTA cannula placements.

#### Exp. 2. Blockade of VTA CRF1 Receptors Does Not Alter Food Self-administration

Intra-VTA antalarmin did not affect food reinforcement in rats. As shown in Fig.1E, rats maintained a similar level of responding for food, regardless of the antalarmin doses they were tested with. A 1-way ANOVA confirmed this observation and revealed no drug effect (F_3,27_ = 0.42; *P* = .66). In addition, intra-VTA antalarmin had no effect on inactive lever pressing (Fig. 1D, F_3,27_ = 1.48; *P* = .27), suggesting antalarmin does not impair general behavior but rather it reduces heroin but not food reinforcement.

#### Exp. 3. Intra-VTA Antalarmin Has No Effect on Locomotor Activity

To test whether antalarmin at the dose range that affects heroin IVSA has any effects on animals’ motor function, we measured locomotor activity under the intra-VTA antalarmin treatment. As shown in Fig. 1F, locomotor activity gradually declined overtime and was not affected by intra-VTA antalarmin. Rats showed similar locomotion, regardless of the antalarmin dose they were treated with. A 2-way ANOVA (time × group) revealed a significant time effect (F_11,336_ = 24.12; *P* = .000) but no group effect (F_3,336_ = 0.13; *P* = .93) and no interaction (F_33,336_ = 0.93; *P* = .58), suggesting that intra-VTA does not produce nonspecific effects on behavior.

#### Exp. 4. VTA CRF1 Receptors on DA Neurons and GluR1-Expressing Neurons Are Upregulated After Heroin IVSA

**Figure 2.**
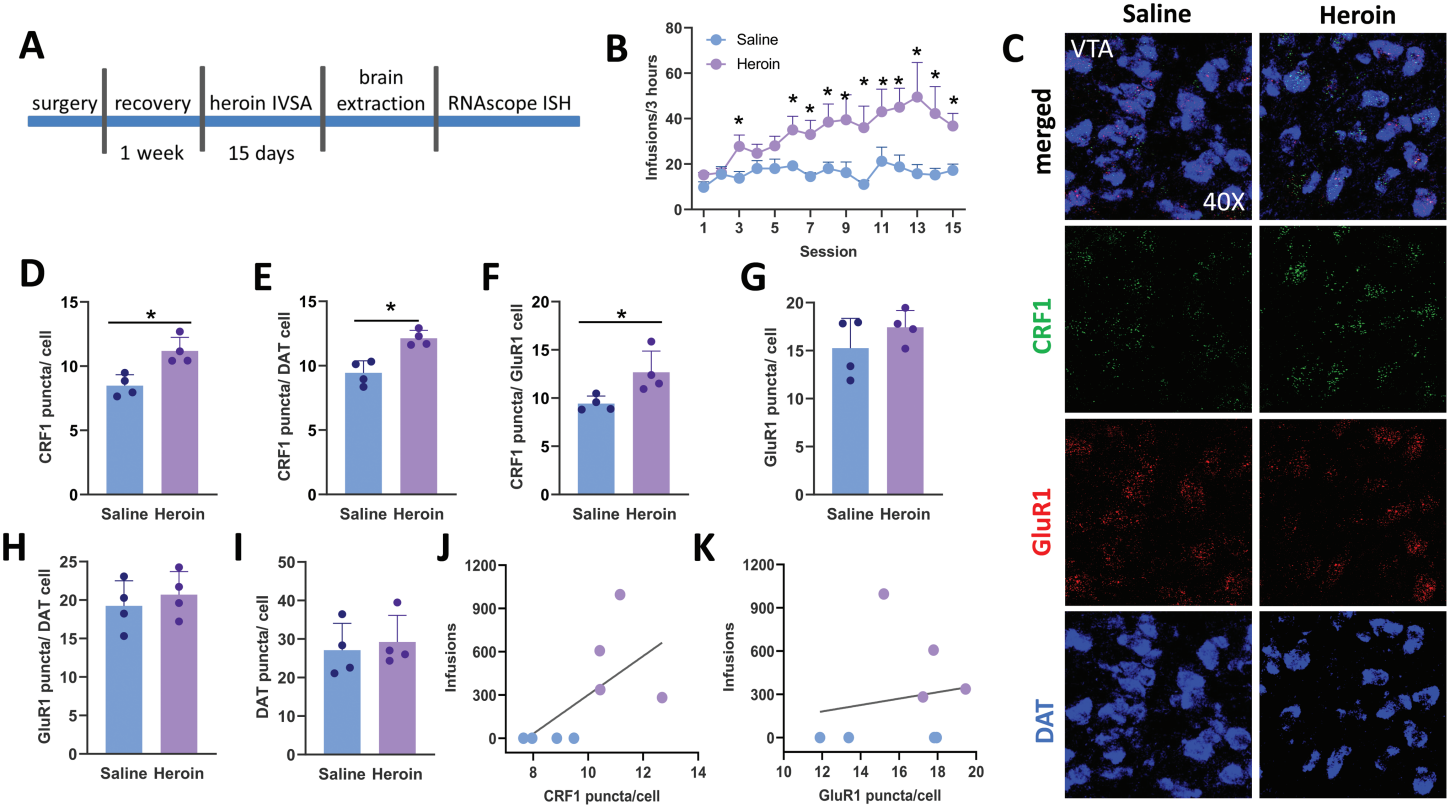
A–K show the data from Exp. 2 investigating neuroadaptations in the VTA after chronic heroin or saline IVSA. (A) The timeline of behavioral assessment and RNAscope ISH assay. (B) Average means of heroin or saline IVSA across 15 sessions, indicating an escalating increase of heroin intake across sessions and significant group differences within each session; *P < .05. (C) Representative images taken at 40× showing colocalization of CRF1, dopamine transporter ( DAT), and AMPA glutametergic receptors 1 (GluR1) mRNA in the VTA. A higher expression of VTA CRF1 mRNA was observed in heroin self-administering rats compared with saline rats. (D) In contrast to saline rats, heroin rats had higher numbers of CRF1 puncta per VTA cells; *P < .05 (E) per DAT cells (*P < .05) and (F) per AMPA GluR1-expressing neurons in the VTA (*P < .05). (G) The AMPA GluR1 mRNA expression in the VTA did not change after heroin IVSA. (H) Similar levels of GluR1 were observed in DAT cells of heroin and saline rats. (J) VTA CRF1 expression significantly correlated with the total number of heroin intake (*P < *.05). (K) There was no correlation between VTA GluR1 mRNA expression and total heroin intake across 15 IVSA sessions.

Next, we assessed whether CRF1 and AMPA (GluR1) receptors in the VTA undergo neuroplastic changes after chronic heroin IVSA. Fig. 2A shows a timeline of behavioral experimentation. Across 15 days, rats self-administered more heroin than saline and showed a steady increase in heroin intake (Fig. 2B). A 2-way ANOVA confirmed this observation revealing a significant phase × dose interaction (F_14,84_ = 1.90; *P* = .037). Tests of simple effects of dose at each session revealed significant group differences (*P < *.05) except in sessions 1, 2, 4, and 5. After 15 days of saline or heroin IVSA, we analyzed *CRF1*, *GluR1*, and *DAT* mRNA expression in the VTA of rats using the RNAscope ISH assay. We were particularly interested in the expression of CRF1 and GluR1 mRNA in DA neurons as both receptors play a role in heroin addiction and their stimulation activates DA neurons. Our analysis revealed that DA and non-DA neurons in the VTA expressed CRF1 and GluR1 receptor mRNA (Fig. 2C). In contrast to saline rats, heroin rats had significantly more CRF1 receptor mRNA puncta per cell (Fig. 2C–D; t_6_ = 3.96*; P* = .007), per DAT cell (Fig. 2C and E; t_6_ = 4.82 *P* = .002), and per GluR1-expressing cells (Fig. 2F; t_6_ = 2.77 *P *=* *.032). As per Pearson correlation, the total number of infusions earned within 15 days significantly correlated with the number of CRF1 mRNA (r = 0.62; *P* = .049; Fig. 2J). However, saline and heroin rats showed a similar expression of GluR1 mRNA per VTA cell (Fig. 2C and G; t_6_ = 1.21; *P* = .269) and per DA cell (Fig. 2C and H; t_6_ = 0.66; *P *=* *.53) and similar expression of DAT mRNA (Fig. 2C and I; t_6_ = 0.428; *P *=* *.683). There was no correlation between the total numbers of heroin infusions and AMPA GluR1 mRNA expression (Fig. 2K; r = 0.57; *P *=* *.354).

Overall, these findings indicate that CRF1 receptors in the VTA are upregulated after chronic heroin IVSA. Such neuroadaptation occurs on DA and non-DA neurons and on GluR1-expressing cells.

#### Exp. 5. Heroin Self-Administration Upregulates CRF1 Receptor Expression in the VTA and NAc

**Figure 3.**
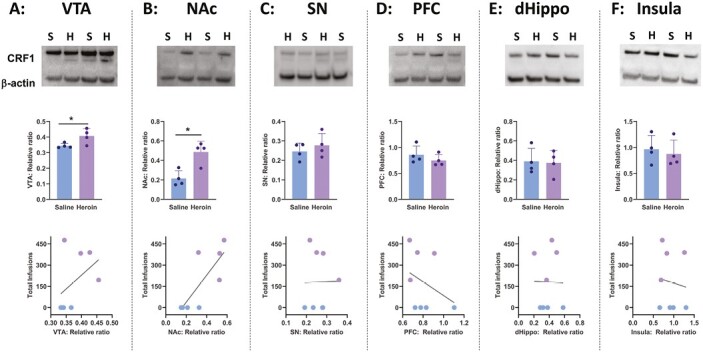
A–F show the data from Exp. 3 investigating corticotropin-releasing factor 1 (CRF1) receptor neuroadaptation after heroin or saline intravenous self-administration (IVSA). Top panels show blot image data from western-blot assay detecting CRF1 (48 kDa) and beta-actin protein (45 kDa). Middle panels show data quantification showing the relative ratio between CRF1 and beta-actin protein expression. Significant upregulation of CRF1 protein was observed in (A) the ventral tegmental area, VTA (*P < .05) and (B) nucleus accumbens, NAc (*P < .05) but not (C) substantia nigra, SN, (D) prefrontal cortex, PFC, (E) dorsal hippocampus, dHippo, or (F) insula. Bottom panels show correlations between relative ratio and heroin intake. A significant correlation was observed between heroin intake and relative ratio of CRF1: beta-actin expression in the NAc (B) but not for other brain regions.

Inspired by these findings, we then used the western-blot assay to assess CRF1 protein expression in the VTA and other brain regions that are known to play a role in heroin IVSA and relapse. Brain regions of interest were VTA, NAc, insula, PFC, dHippo, and SN. Across 15 days, a new set of rats self-administered more heroin than saline infusions (data not shown). This observation was confirmed by a 2-way ANOVA that revealed a significant dose × phase interaction (F_14,84_ = 3.37; *P *=* *.000) and by tests of simple effects of dose at each session, indicating significant group differences in sessions 6–15 (*P < *.05). As indicated in Fig. 3A top and middle panels and by an independent *t* test, heroin rats had significantly higher expression of CRF1 protein in the VTA (t_6_ = 3.47; *P *=* *.048) that moderately correlated with the number of heroin infusions (Pearson correlation: r = 0.43; *P *=* *.13; Fig. 3A bottom panel). Significant upregulation in CRF1 protein expression was also observed in the NAc (Fig.3B top and middle panels; t_6_ = 3.93; *P *=* *.007) that was correlated with the total number of infusions (Fig. 3B bottom panel; Pearson correlation: r = 0.78; *P *=* *.02). After 15 days of heroin IVSA, CRF1 protein level remained unchanged in the SN (Fig. 3C; t_6_ = 0.83; P = .434), PFC (Fig. 3D, t_6_ = 1.06; *P *=* *.324), dHippo (Fig. 3E; t_6_ = 0.16; *P* = .874), and insula (Fig. 3F; t_6_ = 0.47; *P *=* *.650). There was no significant relationship between CRF1 receptor protein expression in these regions (SN, PFC, dHipp, insula) and the total number of heroin infusions (Fig. 3D–F, bottom panels, *P* > .05).

Overall, these data indicate that upregulation of CRF1 receptor protein expression caused by chronic heroin IVSA is region-specific.

#### Exp. 6. 15 Days of EE Fails to Reverse CRF1 Receptor Upregulation in the VTA but Downregulates DAT mRNA

**Figure 4.**
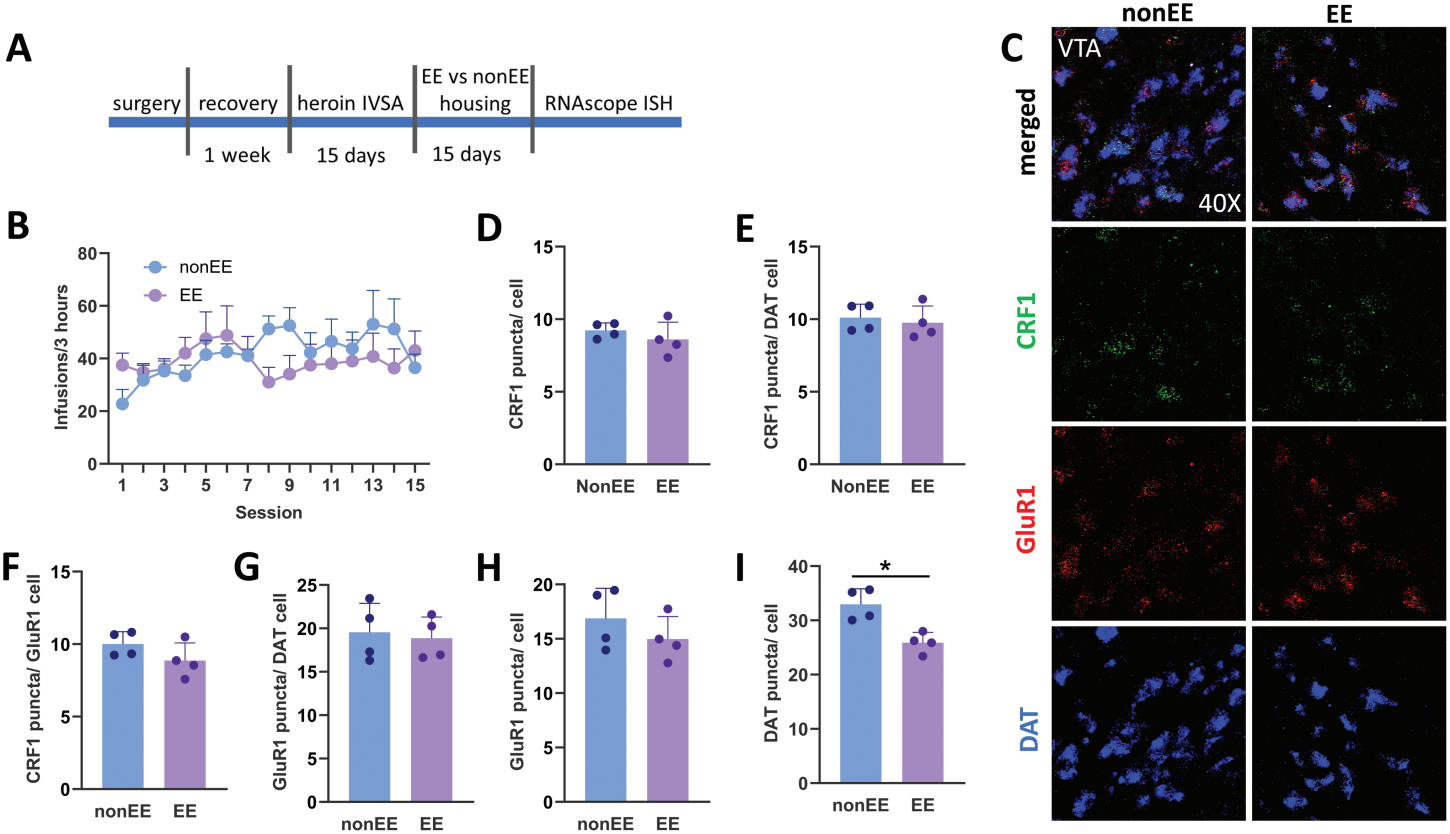
A–I show the data from Exp. 4 investigating the impact of environmental enrichment (EE) on corticotropin-releasing factor (CRF1), AMPA glutamatergic receptor 1 (GluR1) and dopamine transporter (DAT) mRNA in the ventral tegmental area (VTA). (A) A timeline of behavioral assessment, EE, and RNAscope assay. (B) Average means of heroin infusions across 15 sessions, indicating a similar pattern of heroin intravenous self-administration (IVSA) between EE and non-EE groups. (C) Representative images taken at 40× showing CRF1, GluR1, and DAT mRNA expression in EE and non-EE rats. (D) The similar levels of CRF1 mRNA on VTA cells, (E) DAT cells, and (F) GluR1-expressing cells were detected in EE and non-EE rats. (G) There was no significant difference between EE and non-EE groups in the GluR1 mRNA puncta expression per VTA cells and (H) per DA cells. (I) EE exposure downregulated DAT mRNA expression in the VTA (*P < .05).

Lastly, using the RNAscope ISH assay, we assessed whether environmental enrichment (EE) administered after 15 days of heroin IVSA can reverse upregulation of CRF1 receptors in the VTA. Fig. 4A shows a timeline of behavioral experimentation involving 15 days of IVSA, followed by 15 days of EE or non-EE housing. Rats in EE and non-EE showed similar patterns of heroin IVSA, as indicated in Fig. 4B and confirmed by a 2-way ANOVA (F_14,84_ = 1.719; *P *=* *.067). This indicates that groups self-administered similar amounts of heroin before the EE treatment was introduced. After 15 days of EE housing, rats showed a similar level of CRF1 mRNA puncta expression per VTA cell (Fig. 4C-D; t_6_ = 0.96; *P *=* *.378), per DA cell (Fig. 4C and E; t_6_ = 0.47; P = .649), and GluR1-expressing cells (Fig. 4C and F; t_6_ = 1.54; *P *=* *.173) compared with non-EE rats. Similarly, there was no significant difference between EE and non-EE groups in the GluR1 mRNA puncta expression per VTA cell (Fig. 4C and G; t_6_ = 1.10; P = .312) and per DA cell (Fig. 4C and H; t_6_ = 0.33; *P *=* *.745). To our surprise, EE significantly downregulated DAT mRNA expression (Fig. 4C and I; t_6_ = 4.07; *P *=* *.006).

Overall, these findings indicate that 15 days of EE can downregulate DAT mRNA but has no effect on VTA CRF1 and GluR1 expression.

## DISCUSSION

Our major findings in this study are the following: (1) using bilateral microinjections of antalarmin, we demonstrated a causal role for VTA CRF1 receptors in the maintenance of heroin IVSA; unique findings that have not been shown before; (2) we found that intra-VTA antalarmin had no impact on food self-administration nor on locomotor activity; (3) we found that CRF1 receptor mRNA in the VTA are expressed on dopamine and nondopamine neurons and often co-localize with AMPA GluR1 receptors. Using an RNAscope ISH assay, we demonstrated that chronic heroin IVSA leads to upregulation of CRF1 receptors but not of AMPA GluR1 mRNA in the VTA, and this upregulation occurs on dopamine and non-dopamine neurons. We also observed an upregulation of CRF1 receptors on cells expressing AMPA GluR1 receptors; (4) CRF1 receptor protein level in the VTA and NAc is significantly elevated after chronic heroin IVSA, but CRF1 receptors in other key brain regions, including the PFC, insula, SN and dHippo are not; and (5) EE, when implemented after heroin IVSA, does not reverse upregulation of VTA CRF1 receptors but does downregulate DAT mRNA.

CRF is a neuropeptide that mediates hormonal, autonomic, and behavioral responses to stress through stimulation of CRF1 and CRF2 receptors and has been implicated in addiction (Boyson et al., 2011, 2014). There is evidence indicating that CRF within the mesolimbic dopamine system controls stress-related addictive behaviors. For example, infusions of CRF directly into the VTA increase VTA glutamate and dopamine levels and reinstate cocaine taking (Wang et al., 2005), whereas foot shock stress leads to significant increases in extracellular levels of VTA CRF, glutamate and dopamine in cocaine-experienced rats and reinstatement of cocaine seeking (Wang et al., 2005). On the other hand, injections of CRF antagonists or genetic deletion of CRF1 receptors attenuates stress-induced reinstatement of cocaine seeking (Shaham et al., 1998; McFarland et al., 2004; Chen et al., 2014). Electrophysiological recordings also showed that CRF regulates excitability of VTA dopamine neurons (Wanat et al., 2008), often through potentiation of excitatory neurotransmission (Hahn et al., 2009). Of particular interest to our study was the role of VTA CRF1 in heroin IVSA and the impact of heroin IVSA and EE on their expression. We found that microinjections of antalarmin directly into the VTA reduced heroin IVSA in rats, suggesting that these receptors play a critical role in heroin IVSA. This is in line with previous studies indicating that blockade of VTA CRF1 receptors prevents social stress-related escalation of cocaine IVSA (Boyson et al., 2011; 2014). VTA CRF and CRF1 receptors have been implicated in stress-induced drug seeking (Wang et al., 2005; Blacktop et al., 2011; Chen et al., 2014; Vranjkovic et al., 2018). Indeed, chronic exposure to opiates potentiates stress-responses that are controlled by CRF signaling through CRF1 receptors (Contarino and Papaleo, 2005; Baidoo and Leri, 2022). However, new findings also indicate that optogenetic stimulation of CRF-containing neurons in the central amygdala or NAc, but not bed nucleus of stria terminalis, enhances break points for cocaine in self-administering transgenic CRH-cre rats without causing significant stress responses (Baumgartner et al., 2022).

As we demonstrate here a causal role of VTA CRF1 receptors in heroin IVSA, it is important to emphasize that CRF stimulates both CRF1 and CRF2 receptors. Thus, it is conceivable that VTA CRF2 receptors might also be important players in heroin taking and seeking, given the fact that prior studies showed the involvement of VTA CRF2 receptors in stress-induced drug seeking (Wang et al., 2007) and ethanol drinking (Albrechet‐Souza et al., 2015). Our future studies will assess whether heroin IVSA requires stimulation of VTA CRF2 receptors and how their expression is affected by chronic heroin exposure.

It is also important to point out that intra-VTA antalarmin did not affect food self-administration, which suggests that stimulation of VTA CRF1 receptors is not necessary for food reinforcement. This is in line with a previous study demonstrating that intra-VTA antalarmin (500 ng) failed to alter food-reinforced lever pressing during a 30-minute session and under a higher reinforcement schedule (FR4) (Blacktop et al., 2011). In addition, intra-VTA antalarmin did not have a significant effect on inactive lever pressing or spontaneous locomotor activity, suggesting that intra-VTA antalarmin reduces heroin-reinforced behavior rather than nonspecific behavior.

Our work also indicates that 15 days of heroin IVSA leads to upregulation of VTA CRF1 receptors, particularly on dopamine neurons. Dopamine neurons also express AMPA GluR1 receptors but their expression was not impacted by chronic heroin IVSA. Thus, these findings suggest that a unique relationship between CRF-dopamine and glutamatergic neurotransmission becomes dysregulated after chronic heroin exposure, primarily through upregulation of VTA CRF1 receptors on DAT and AMPA GluR1-expressing neurons. We also found heroin-induced upregulation in the NAc, a brain region implicated in opioid reward, reinforcement and relapse. Previous studies showed that CRF mRNA within the central amygdala and hypothalamus are upregulated in opioid-dependent animals (McNally and Akil, 2002). To our surprise, we found no change in CRF1 protein level in the PFC, insula, dHippo, and SN. As mentioned previously, several studies provided compelling evidence that these brain regions are implicated in opioid-driven behaviors. Thus, it appears that the effect of heroin IVSA on CRF1 receptor expression seems to be region-specific and CRF1 receptor neuroadaptations occur in the regions controlling the rewarding and reinforcing effects of drug IVSA and mediated by dopamine signaling.

To our great disappointment, we found no evidence that heroin-induced neuroadaptations within the reward DA system can be reversed by EE. Before EE was introduced, EE and non-EE rats showed a similar pattern of heroin intake. Rats housed in EE for 15 days, introduced after heroin IVSA training, showed a similar level of CRF1 mRNA in the VTA as control non-EE rats. These findings contradict previous studies indicating that EE can reverse some of drug- or alcohol-induced neuroadaptations within the mesocorticolimbic system (Lynch et al., 2010; Solinas et al., 2010; Abel et al., 2019; Galaj et al., 2020a). EE can reverse the effects of stress on behavioral responses and associated CRF signaling (Francis et al., 2002; Sztainberg et al., 2010; Fan et al., 2021). Specifically, EE can decrease anxiety-like behaviors in mice and reverse the upregulation of CRF1 mRNA level in the basolateral amygdala caused by chronic stress (Sztainberg et al., 2010; Hendriksen et al., 2012). Furthermore, CRF signaling is recruited with repeated drug use in a manner that increases susceptibility to stress-induced drug seeking (Zorrilla et al., 2014), but EE dampens the response of CRF to stress and produces anxiolytic effects. Indeed, previous studies have shown that EE can reduce stress-induced reinstatement of drug seeking (Chauvet et al., 2009). In our hands, EE failed to reverse upregulation of VTA CRF1 receptors. Perhaps, 15 days of EE is not sufficient to restore CRF1 signaling. We previously found behavioral effects of EE when implemented for 15 (Barrera et al., 2021), 30 (Galaj et al., 2016), or 60 days (Galaj et al., 2017). But based on the current findings, it appears that 15 days is not sufficient to restore the CRF system after chronic opioid IVSA. In addition, others have shown that the hyper-response of hypothalamo-pituitary-adrenal system to stress but not the upregulation of CRF mRNA in the PVN of hypothalamus induced by maternal separation can be reversed by EE (Francis et al., 2002). As the authors eloquently pointed out this suggests that EE may lead to a functional reversal of the effects of stress through compensation for, rather than reversal of, the neural effects of such stress. However, we found that EE downregulates the level of DAT mRNA in VTA neurons. This finding is in line with previous reports that EE decreases DAT cell surface expression in the PFC (Zhu et al., 2005) and DAT function (Neugebauer et al., 2004), suggesting that EE might adjust dopamine clearance that might be dysregulated with chronic heroin IVSA. Thus, we believe that the lack of EE effect on CRF1 receptor level indicates that EE-induce downregulation of VTA DAT is unlikely a CRF1 receptor dependent process. Perhaps, EE produces anxiolytic effects by reducing CRF level in the VTA and NAc or causing neuroadaptations at the CRF2 receptor levels. Sensory inputs from grooming and handling (considered forms of EE) can restore CRF2 mRNA expression in the ventromedial hypothalamus and basolateral amygdala of pups deprived of maternal care (Eghbal-Ahmadi et al., 1999). Thus, we speculate that EE can restore the CRF-dopamine-glutamate interaction through non-CRF1 receptor neuroadaptation.

Although this is beyond the scope of this study, it is important to assess potential sex differences in heroin or EE effects on behavior and neuronal adaptation. In our previous studies, we demonstrated that EE is effective at facilitating abstinence in a conflict paradigm and heroin seeking in the incubation paradigm, regardless of sex. We found no sex differences in EE effects on drug seeking (Barrera et al. 2021 and 2023). Our future studies, which are currently underway, will assess the impact of chronic heroin exposure on female CRF system. Females are more prone to stress and anxiety than males; thus, we expect to observe a higher impact of heroin-related stress on dysregulation of the CRF system, including VTA CRF1 and CRF2 receptors. Previous studies point out significant sex differences in CRF mRNA expression in the bed nucleus of stria terminalis and paraventricular nucleus after cocaine exposure (Connelly and Unterwald, 2020). Nicotine exposure also decreases acccumbal CRF1 expression in females, most likely due to elevated CRF expression (Torres et al., 2015).

In conclusion, through a series of experiments we have demonstrated that VTA CRF1 receptors play a critical role in the maintenance of heroin IVSA, which in turn leads to increases in CRF1 expression in the VTA and NAc, 2 brain regions strongly involved in the rewarding and reinforcing effects of opioids. Heroin IVSA has little, if at all, impact on CRF1 receptor protein expression in the PFC, insula, SN and dHippo. EE when implemented after heroin IVSA fails to reverse heroin-induced upregulation of VTA CRF receptors while it downregulates DAT mRNA in VTA neurons, perhaps due to its anxiolytic effects. The ineffectiveness of EE on heroin-induced upregulation of CRF1 gene expression suggests that dysregulation of VTA CRF system is among the long-lasting neuroadaptations that occur with chronic drug use.

## Data Availability

We are happy to share any protocols or programs that we used to collect these data. The data will be made available upon direct requests to the corresponding authors.
